# The Zinc Transporter, *Slc39a7* (*Zip7*) Is Implicated in Glycaemic Control in Skeletal Muscle Cells

**DOI:** 10.1371/journal.pone.0079316

**Published:** 2013-11-12

**Authors:** Stephen A. Myers, Alex Nield, Guat-Siew Chew, Mark A. Myers

**Affiliations:** 1 Collaborative Research Network and the School of Health Sciences, University of Ballarat, Mount Helen Campus, Victoria, Australia; 2 School of Health Sciences, University of Ballarat, Mount Helen Campus, Victoria, Australia; CNRS UMR7275, France

## Abstract

Dysfunctional zinc signaling is implicated in disease processes including cardiovascular disease, Alzheimer's disease and diabetes. Of the twenty-four mammalian zinc transporters, ZIP7 has been identified as an important mediator of the ‘zinc wave’ and in cellular signaling. Utilizing siRNA targeting *Zip7* mRNA we have identified that *Zip7* regulates glucose metabolism in skeletal muscle cells. An siRNA targeting *Zip7* mRNA down regulated *Zip7* mRNA 4.6-fold (p = 0.0006) when compared to a scramble control. This was concomitant with a reduction in the expression of genes involved in glucose metabolism including *Agl*, *Dlst*, *Galm, Gbe1, Idh3g, Pck2*, *Pgam2, Pgm2, Phkb, Pygm, Tpi1, Gusb and Glut4*. Glut4 protein expression was also reduced and insulin-stimulated glycogen synthesis was decreased. This was associated with a reduction in the mRNA expression of *Insr, Irs1* and *Irs2*, and the phosphorylation of Akt. These studies provide a novel role for *Zip7* in glucose metabolism in skeletal muscle and highlight the importance of this transporter in contributing to glycaemic control in this tissue.

## Introduction

Cellular zinc storage, release and distribution are controlled by a family of zinc transporters and metallothioneins. In mammals two families of zinc transporters exist: the zinc efflux (Slc30/ZnT) and the zinc influx (Slc39/ZIP) proteins [1]. ZnT proteins transport zinc out of the cell or into subcellular compartments in the presence of high cytoplasmic zinc. In contrast, ZIP proteins transport zinc into the cell or out of subcellular compartments when cytosolic zinc is low or depleted [2].

There is increasing interest in the importance of zinc transporters in diseases associated with dysfunctional cellular signaling. In particular, a significant role for these transporters in maintaining essential glucose and lipid metabolism has been identified. For example, in myocytes isolated from the femoral muscle of ZnT7 knockout mice, a reduction in insulin signaling pathway activity was observed [3]. The ZnT7 null mice were susceptible to diet-induced glucose intolerance and insulin resistance and this was associated with a decrease in the expression of the insulin receptor, insulin receptor substrate 2 and Akt1 [3]. ZnT3, ZnT5 and ZnT8 gene expression are differentially regulated by glucose in INS-IE cells, and streptozotocin-treated ZnT3 null mice have decreased insulin gene expression and insulin secretion that resulted in hyperglycemia [4]. Moreover, ZnT8 plays a critical role in the synthesis and secretion of insulin and therefore represents a pharmacological target for treating disorders of insulin secretion including diabetes [5].

Zinc mediates its effects through two mechanisms; early zinc signaling (EZS) and late zinc signaling (LZS) [6]. LZS occurs several hours after an extracellular signaling event and depends on changes in the expression of zinc-related molecules such as zinc transporters and metallothioneins [6], [7]. In contrast, EZS occurs minutes after an extracellular stimulus and does not involve transcriptional-dependent changes [6], [7]. Zinc signaling mechanisms are involved in eliciting an increase in intracellular zinc concentrations − the ‘zinc wave’ phenomenon [8]. Thus, in this situation zinc acts as a second messenger that activates pathways associated with cellular signaling. In fact, zinc has been categorized as an insulin-mimetic with several groups examining the role of its mimetic activity on glucose [9]–[13] and lipid [13], [14] metabolism. In this context ZIP7 has been identified as a key zinc transporter implicated in the “zinc wave” and is suggested to be a “gatekeeper” of cytosolic zinc release from the ER [8]. Endogenous ZIP7 is predominately localized to the Golgi apparatus [15], the ER [16], or both [17] and has been implicated in breast cancer progression [8], [17], [18]. Studies in tamoxifen-resistant MCF-7 breast cancer cells identified that ZIP7 was responsible for activation of multiple tyrosine kinases that are implicated in the aggressive phenotype of tamoxifen-resistant breast cancer [8], [19], [20]. Recent evidence in MCF7 cells suggests that ZIP7 is phosphorylated by CK2 and is associated with the regulated release of zinc from intracellular stores to phosphorylate kinases implicated in cell proliferation and migration [8].

Given the role of ZIP7 in modulating zinc flux, and the role of zinc as an insulin mimetic in cellular processes, we propose that ZIP7 may also be implicated in metabolic processes associated with glycaemic control. Here we report evidence for a novel role for *Zip7* in modulating glycaemic control in skeletal muscle cells. We find that the attenuation of *Zip7*mRNA in C2C12 skeletal muscle cells modulates genes involved in carbohydrate metabolism and glycogen synthesis. These studies demonstrate a previously unprecedented role for *Zip7* in regulating glycaemic control in skeletal muscle and provide a platform to further explore the potential of this transporter in skeletal muscle insulin resistance.

## Materials and Methods

### Cell culture

Proliferating mouse C2C12 myoblasts in all experiments were cultured and maintained in DMEM supplemented with 10% Fetal Bovine Serum and physiological zinc concentrations (20 µM ZnSO_4_), (Life Technologies, Mulgrave, Victoria, Australia). Differentiation of myoblasts into post-mitotic, multi-nucleated myotubes was induced by mitogen withdrawal (i.e. DMEM supplemented with 20 µM Zn SO_4_ and 2% horse serum for three days). Assessment of the muscle-specific, contractile and metabolic C2C12 muscle phenotype was assessed by measuring the expression of markers of differentiation and metabolic processes as previously described [21]. The time course experiments on differentiated C2C12 skeletal muscle cells were performed over 60 minutes in the presence of 10 nM insulin, 20 µM ZnSO_4_ and 10 µM pyrithione (see Figures S1 and S2).

### RNA Extraction and cDNA Synthesis

Mouse quadriceps muscle was a kind gift from Dr. Paul Lewandowski, Deakin University, Australia with approval from the Deakin University Animal Welfare Committee (A37/2007). Total RNA was extracted from C12C2 cells and C57Bl/6J mouse quadriceps using TRI-Reagent (Sigma-Aldrich, Castle Hill, NSW, Australia) according to the manufacturer's protocol. Total RNA was then treated with 2 U of DNase1 for 30 min at 37°C followed by purification of the RNA through an RNeasy purification column system (Qiagen, Chadstone, Victoria, Australia). RNA quantity and quality was measured using a Nanodrop spectrophotometer (Thermo Scientific, Scoresby, Victoria, Australia). A High Capacity cDNA Synthesis kit was used to synthesize cDNA from 2 µg of total RNA using random hexamers according to the manufacturer's instructions (Life Technologies). The cDNA was diluted to 400 µl in nuclease-free water and stored at −20°C.

### Mouse glucose metabolism and zinc transporter arrays

The Mouse Glucose Metabolism RT^2^ Profiler PCR Array was purchased from SA Biosciences, Qiagen; Catalogue No. 330321 PAMM-006ZA. This array profiles the expression of 84 genes involved in the regulation and enzymatic pathways of glucose and glycogen metabolism (Table S1). The Zinc Transporter RT^2^ Profiler Custom PCR Array (Qiagen) contained the genes for the two zinc transporter families, *Slc30a1-10* and *Slc39a1-14*.

### cDNA synthesis of RT^2^ mouse glucose metabolism and zinc transporter PCR array

cDNA synthesis using the RT^2^ First Strand Kit was performed as described by the manufacturer (Qiagen). Briefly, potential genomic DNA was eliminated from 500 ng of total RNA using buffer GE at 42°C for 5 min. The RNA was then reverse transcribed in a total of 20 µl reaction volume for 15 mins at 42°C then the reaction was stopped by heating the sample at 95°C for 5 min.

### Quantitative Real-time PCR

Quantitative PCR (qPCR) was performed on a RealPlex PCR detection system (Eppendorf, North Ryde, New South Wales, Australia) in triplicate on at least three independent RNA preparations. Target cDNA levels were analyzed in 10 µl reactions with SensiMix SYBR No-ROX (Bioline, Alexandria, New South Wales, Australia). Primers (GeneWorks, South Australia, Australia) for markers of skeletal muscle cell differentiation and metabolism, Myogenin, *Tnni1*, *Tnni2*, *Abca1*, *Fabp3* and *Srebp-1c* (Table S2) have been previously described [21]–[23]. Other primers (Table S2) for the amplification of target gene sequences were designed using the NCBI Primer Blast Tool http://www.ncbi.nlm.nih.gov/tools/primer-blast/index.cgi, with the exception of *Irs1* (PrimerBank ID: 29825829a1), *Irs2* (PrimerBank ID: 3661525a1) and *Insr* (PrimerBank ID: 6754360a1) which were obtained from the PrimerBank Database http://pga.mgh.harvard.edu/primerbank/index.html [24]–[26]. Note: all primers were rigorously analyzed by BLAST for target gene specificity and designed to be genomic resistant (i.e. at least one primer crossed an exon-exon boundary). qPCR was performed using 4 µl of cDNA template (20 ng) and 40 cycles of 95°C for 15 seconds, 57°C for 15 seconds and 72°C for 20 seconds. The relative level of target gene expression was normalized to *Gapdh*, or eukaryotic elongation factor 2 (*Eef2*) as described in the results section and associated errors were calculated using the guidelines described by Bookout and Mangelsdorf [27].

### Primer design to detect endogenous and exogenous pCMV-Zip7

Primers were designed to specifically target endogenous and exogenous *Zip7* mRNA. For the pCMV-Zip7 overexpression plasmid, we placed the forward primer on the *Zip7* mRNA sequence and the reverse primer on the plasmid C-myc tag (see Table S2). For the specific amplification of endogenous *Zip7* we designed primers on the 5′UTR. This region is omitted on the pCMV-*Zip7* expression plasmid.

### Quantitative Real-time PCR of RT^2^ mouse glucose metabolism and zinc transporter PCR array

The qPCR for the RT^2^ glucose metabolism and zinc transporter arrays were performed as outlined in the guidelines supplied by the manufacturers (Qiagen). Briefly, cDNA (102 µl) from the RT^2^ First Strand Kit was added to 1350 µl of 2 x RT^2^ SYBR Green and 1248 µl of H_2_O. To each well of the glucose metabolism array 96-well plate, 25 µl of sample was added. qPCR was performed on a RealPlex PCR detection system (Eppendorf) using 45 cycles of amplification consisting 95°C for 15 seconds and 60°C for 1 minute.

### Transient transfections of siRNA molecules

The transient transfection of siRNA molecules *Zip7* (Catalogue No: AM16708), *Zip1* (Catalogue No: AM16708), *Gapdh* (Catalogue No. 4390771) and the scramble control (Catalogue No: AM4635) (Life Technologies) were performed using RNAiMAX reagent as instructed by the manufacturer (Life Technologies). Briefly, C2C12 cells were transfected in 6-well dishes with 10 nM of siRNA molecule or the scramble control in RNAiMAX reagent. The cells were subsequently maintained in 2% horse serum and differentiated over three days and collected in 1 ml of TRI-Reagent per three wells for RNA extraction or 1 ml of RIPA Buffer (Thermo Scientific) (containing Halt Protease and Phosphatase Inhibitor Cocktail; Thermo Scientific) for protein analysis.

### Zip7 overexpression plasmid and transient transfection in C2C12 skeletal muscle cells

A full-length mouse cDNA Zip7 expression plasmid was obtained from Origene Technologies, Inc (Clone ID MR216531; Rockville, MD). The pCMV control plasmid was created by excision of the full-length Zip7 gene by restriction digest at enzyme sites *Sgf1* and *Mlu1* followed by end-filling and blunt-end ligation. Briefly, 1 µg of pCMV-Zip7 plasmid was digested in the presence of 10X Fast Digest Green Buffer and 1 unit of *Sgf1* and *Mlu1* restriction enzymes (Fermentas, Thermo Scientific) for 5 min at 37°C. For the end-filling, approximately 1 µg of pCMV plasmid DNA was incubated with 0.5 mM dNTPs and 1 unit of Klenow fragment and incubated at 30°C for 15 minutes. The pCMV control plasmid was circularized in the presence of 2 µl of 10X T4 DNA ligase, 100 ng of plasmid vector and 1 µl of T4 DNA ligase and incubated at 4°C for approximately 16 h.

The pCMV-Zip7 and pCMV control plasmid were transiently transfected into C2C12 skeletal muscle cells using Lipofectamine 2000 (Life Technologies) as instructed by the supplier. Briefly, C2C12 skeletal muscle cells were grown to 80% confluence and 4 µg of pCMV-Zip7 and the pCMV control vector were mixed with 5 µl of Lipofectamine 2000 and 500 µl of optimen. Following a 20 min incubation at RT, the Lipofectamine-DNA reagent was pipetted onto the C2C12 cells contained in a 6-well plate and supplemented with 1.5 ml of differentiation media. The cells were differentiated for 72 hr and subsequent RNA and protein was extracted as described below.

### Protein Extraction and Western Blot

Total cellular protein from the scramble control and the siRNA-*Zip7* transfected C2C12 cells was isolated by scraping cells with RIPA buffer (contains protease and phosphatase inhibitor cocktail) then the samples were place on ice for 1 hr with constant vortexing every 10 mins. The cells were then sonicated for 5 second pulses for 30 seconds at 50% duty followed by boiling for 10 min. The protein samples were centrifuged at 13,000 rpm for 5 min and the supernatant collected. Total protein concentration was measured using a BCA kit (BIORAD, Gladesville, New South Wales) as outlined by the manufacturer's instructions.

Total soluble protein (100 µg) from the scramble control and siRNA-*Zip7* transfected C2C12 cell lines was resolved on a 4–15% SDS-PAGE gradient gel (BIORAD) and transferred to a nitrocellulose membrane. The membranes were blocked overnight in 5% skim milk in TBS-Tween 20 followed by an overnight incubation with either Glut4 (Cell Signaling, 1∶2000; Catalog No: 2213), Gapdh (1∶5,000; Catalog No: sc-48167) (Santa Cruz Biotechnology, Santa Cruz, CA), Akt (Cell Signaling 1∶5000 Catalog No:9272), pAkt (Cell Signaling, 1∶5000, Catalog No: 4058) antibodies. Following 4×15 minute washes the membrane was incubated with either anti-mouse HRP (Cell Signaling, Catalog No: 7076) for Glut4 (1∶5000); anti-Rabbit HRP (Cell Signaling, Catalog No:7074) for Akt and pAkt (1∶5000), and anti-goat HRP (Santa Cruz; Catalog No: sc-2020) for Gapdh (1∶5000) for 1 hr at RT. Immunoreactive signals were detected using enhanced chemiluminescence SuperSignal West Pico Substrate (Pierce) and visualized by autoradiography or a UVITEC Alliance digital imaging system (Thermo Fisher Scientific, Victoria Australia). Note: to assess protein loading consistency, the membranes were stripped with Restore Plus Western Blot Stripping Buffer (Thermo Fisher) by incubating the membrane in the buffer for 15 min at RT. Membranes were subsequently washes in TBS-Tween and blocked with 5% skim milk before adding the primary antibody.

### Glycogen synthesis assay

The glycogen synthesis assay was performed as described by the manufacturer's (BioVision, Life Research, Scoresby Victoria, Australia). Briefly, C2C12 cells were transfected with siRNA-*Zip7* and the scramble control as described above. Following differentiation, C2C12 cells were treated with 10 nM insulin for 60 minutes. Cell lysates were collected in 200 µl of dH_2_O on ice and homogenates were boiled for 5 min to inactivate enzymes. Samples were then centrifuged at 13000 rpm for 5 min and the supernatant was collected. Samples were then prepared by performing hydrolysis of glycogen to glucose and then mixed with OxiRed probe to generate colour (λ_max_ = 570 nm). Note: a glucose control was also performed in the absence of glucoamylase to determine background glucose levels. These were subsequently subtracted from the glycogen readings. The glycogen concentration in the samples was calculated by C =  Ay/Sv where Ay is the amount of glycogen (µg) in the sample as determined from a standard curve and Sv is the sample volume (µl).

### Statistics

Data obtained from individual qPCR was assessed by a Student's unpaired t-test on at least three independent biological replicates. Statistical significance was denoted as the average ± standard deviation of the mean. Data was considered statistically significance when the *P* value was ≤0.05. *P<0.05; **P<0.01 and ***P<0.001. The analysis of the gene arrays was performed with the RT^2^ Profiler PCR Array Data Analysis Software v3.5 (SA Biosciences).

## Results

### The Slc39a (Zip) zinc transporters are differentially expressed in C2C12 skeletal muscle cells and mouse quadriceps

To determine the expression levels of the *Slc39a* zinc transporter family in mouse C2C12 skeletal muscle cells and mouse quadriceps we utilized a custom zinc gene array (SABiosciences, Qiagen) with primer sequences that are specific for the *Slc39a (Zip)* mouse zinc transporter genes (i.e. *Slc39a1-14*). Quantitative real-time PCR (qPCR) was performed and the expression of each zinc transporter transcript was measured relative to the ‘housekeeping gene’, *Gapdh*.

**Figure 1:**
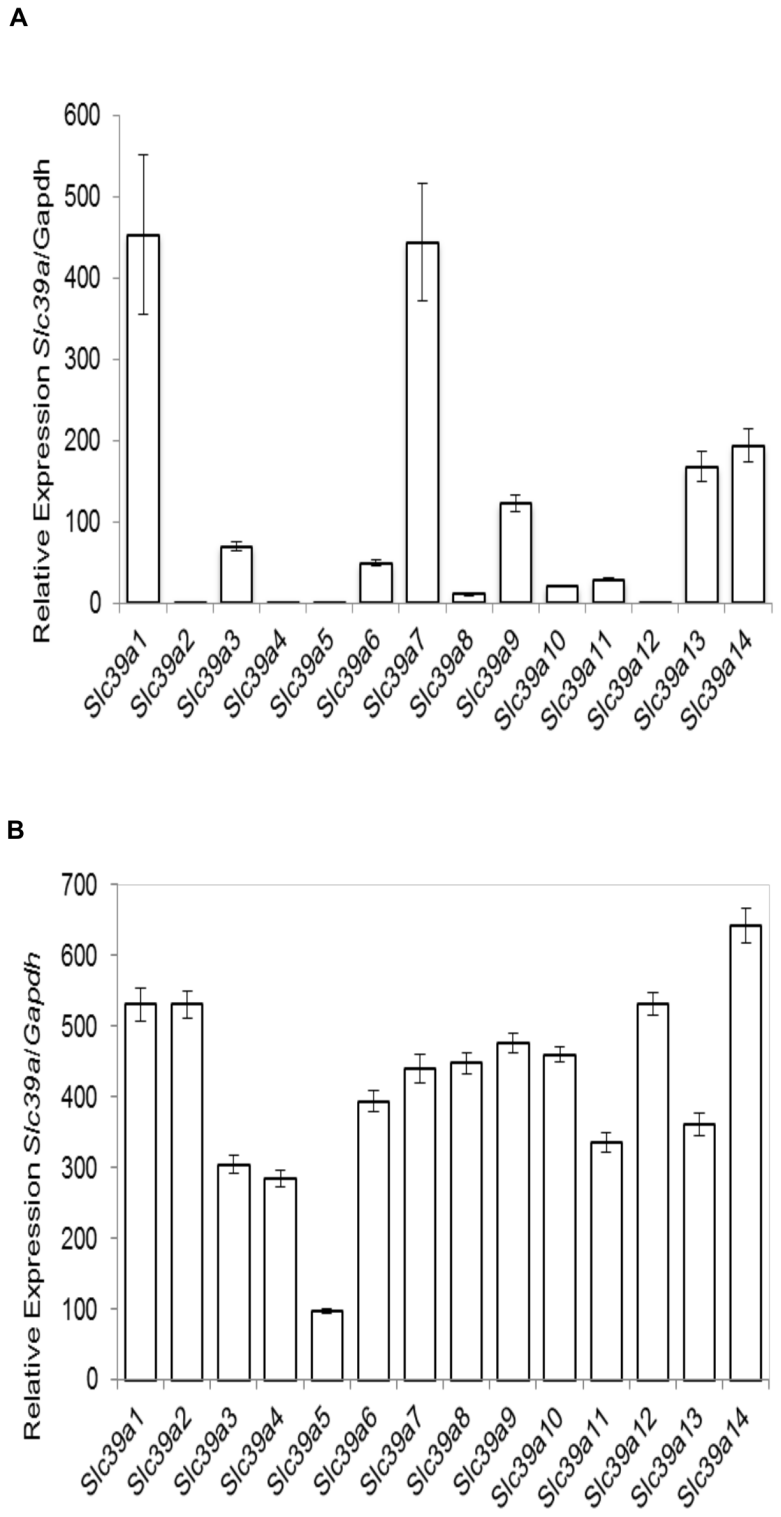
mRNA expression of the zinc transporters Slc39a (Zip) relative to the housekeeping gene, Gapdh in mouse C2C12 skeletal muscle cells and mouse quadriceps. A–B): *Slc39a1-14* mRNA expression in C2C12 cells and mouse quadriceps, respectively. Error bars indicate ± SD from three independent biological samples.

The zinc transporters *Slc39a1 and Slc39a7* were highly expressed in C2C12 skeletal muscle cells (Figure 1A). Lower levels of expression were observed for *Slc39a3, 6, 9, 10, 11, 13* and *14*. Minimal or no expression was observed in *Slc39a2, 4, 5, 8*, and *12* (Figure 1A). In mouse quadriceps we observed high levels of expression for all of the *Slc39a* transporters with the exception of *Slc39a5* (Figure 1B).

### Slc39a7 (Zip7) is expressed during C2C12 skeletal muscle differentiation

**Figure 2:**
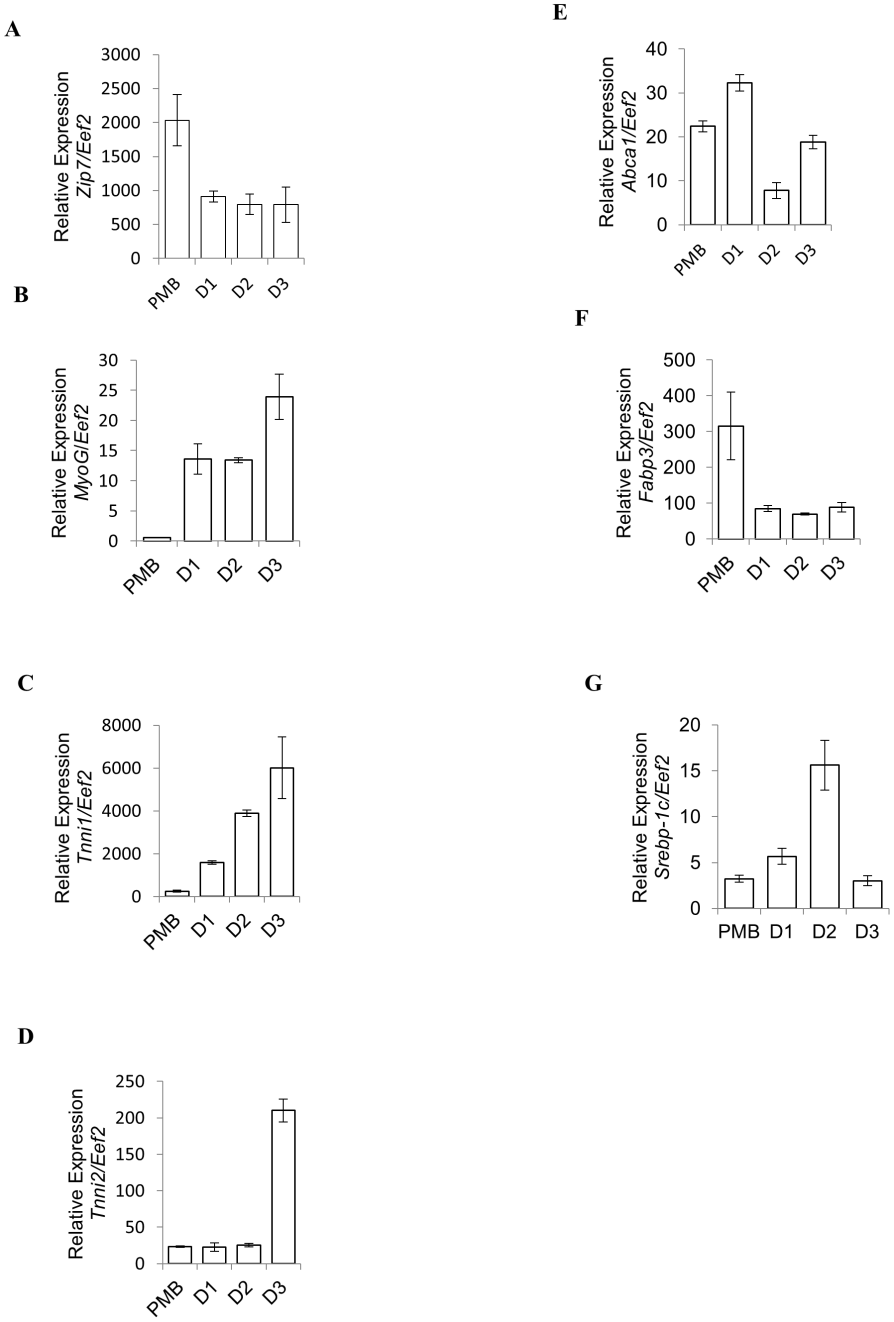
Relative expression of Slc39a7 (Zip7) and markers of skeletal muscle differentiation in C2C12 cell lines. A). *Slc39a7* (*Zip7*) expression relative to *Eef2*, B–D). Markers of skeletal muscle differentiation: myogenin (*MyoG*) and the troponins 1 and 2 (*Tnni1* and *Tnni2*), respectively. E–G). Markers of metabolism: ATP-binding cassette transporter protein 1 (*Abca1*), fatty-acid binding protein 3 (*Fabp3*) and sterol regulatory element binding protein 1c (*Srebp-1c*), respectively. PMB =  proliferating myoblasts; D1–3 =  day 1 to day 3 of differentiation of myotubes, respectively. Error bars indicated the ± SD from three independent biological samples.

We were most interested in *Slc39a7* (*Zip7*) as this transporter is predominately localized to the ER and the Golgi apparatus and is suggested to be involved in the ‘zinc wave’ and associated cellular signaling [8]. Accordingly, to elucidate the role of *Zip7* in skeletal muscle we initially investigated the expression profile of this zinc transporter relative to *Eef2* in the mouse C2C12 myoblast cell line. Proliferating myoblasts can be induced to biochemically and morphologically differentiate into post-mitotic multinucleated myotubes by mitogen withdrawal. This transition from a non-muscle phenotype to a contractile phenotype is associated with the activation and repression of a structurally diverse group of genes responsible for contraction and the extreme metabolic demands placed on this tissue [21]. During this period of differentiation, we observed that *Zip7* mRNA is highly expressed in proliferating myoblasts and was constitutively expressed during skeletal muscle cell differentiation when normalized to *Eef2* (Figure 2A).

In order to assess the differentiation status of the C2C12 cells and to demonstrate that they had acquired a differentiated, contractile and metabolic phenotype, qPCR was performed on the marker genes myogenin (*MyoG*), a gene that encodes the hierarchical basic helix loop regulator and is specifically required for differentiation [28], the slow twitch (type I) and the fast twitch (type II) isoforms of the contractile protein troponin I (*Tnni1* and *TnniII*), and the metabolic genes *Abca1* (ATP-binding cassette proteins), *Fabp3* (fatty acid binding protein 3) and *Srebp1c* (sterol regulatory binding element protein). Expression of both *MyoG* and the contractile protein genes (type I and II, *Tnni1* and *Tnni2*, respectively) were dramatically increased and confirmed the differentiation of the myoblast C2C12 skeletal cell line to the myotube phenotype (Figure 2B–D). Additionally, genes involved in lipid metabolism (*Abca1* and *Srebp1c*) (Figure 2E and 2G) were also induced while *Fabp3* was downregulated during muscle differentiation (Figure 2F) which is consistent with previous studies [22], [29]–[31] and confirms that the muscle cells had acquired the appropriate contractile and metabolic phenotype.

### siRNA-Zip7 Expression Represses Endogenous Zip7 mRNA in Skeletal Muscle Cells

To elucidate the biological role of *Zip7* in the context of glucose metabolism we selectively ablated the expression of this transporter in C2C12 skeletal muscle cells utilizing a siRNA-*Zip7* molecule. An siRNA targeting mouse *Gapdh* and a scramble sequence that contains no known homology to the mouse, rat or human genome were utilized as controls. The siRNA-*Gapdh* was used to determine the robustness of the transfection and the ability to successfully attenuate a specific target gene that is constitutively expressed. Accordingly, C2C12 cells were transfected with the scramble control, siRNA-*Gapdh* or the siRNA-*Zip7* and subsequently differentiated for three days.

**Figure 3:**
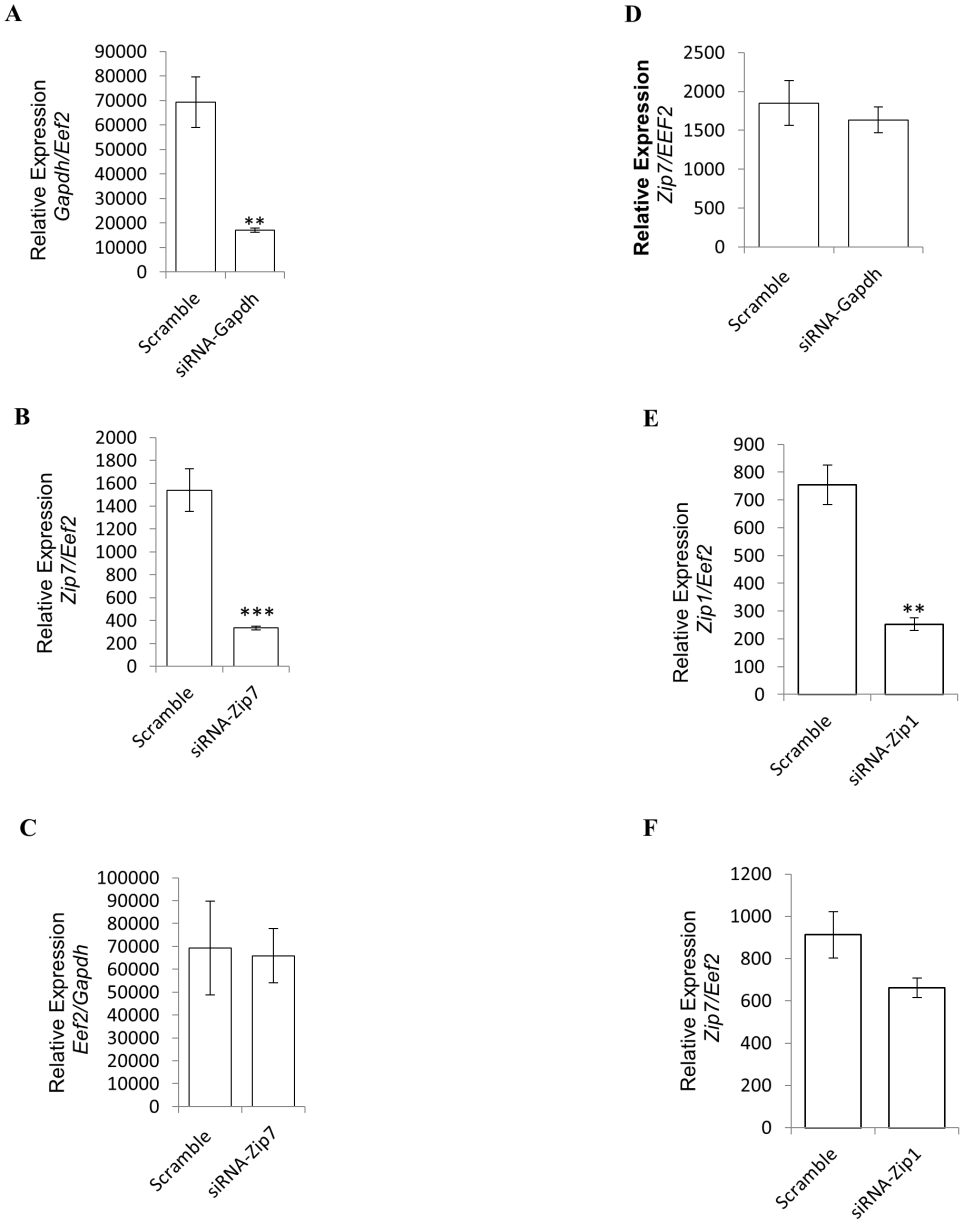
Zip7 mRNA is attenuated by si-RNA-Zip7. Relative expression of *Gapdh, Zip7, Zip1* and *Eef2* in the scramble control and corresponding siRNA cells, respectively. A). *Gapdh* relative to *Eef2* in siRNA-*Gapdh* cells B). *Zip7* relative to *Eef2* in siRNA-*Zip7* cells C). *Eef2* relative to *Gapdh* in siRNA-*Zip7* cells D). *Zip7* relative to *Eef2* in siRNA-*Gapdh* cells E). *Zip1* relative to *Eef2* in siRNA-*Zip1* cells, and F). *Zip7* relative to *Eef2* in siRNA-*Zip1* cells. Error bars indicated the ± SD from three independent biological samples. **P<0.01, ***P<0.001.

Initially, we aimed to validate the specificity and robustness of the siRNA transfection in C2C12 cells by transfecting an siRNA-*Gapdh* to determine transfection efficacy and siRNA specificity. We identified a significant reduction in *Gapdh* mRNA (4-fold, p = 0.0023) in the siRNA-*Gapdh* transfected cells compared to the scramble control (Figure 3A). We then transfected C2C12 skeletal muscle cells with an siRNA targeting *Zip7* mRNA. Quantitative PCR was then performed to measure the expression levels of endogenous *Zip7* relative to *Eef2* in RNA isolated from the scramble control and *Zip7* transfected cell lines. We observed a significant reduction in the mRNA levels of *Zip7* (4.6-fold, p = 0.0006) when compared to the scramble control (Figure 3B).

**Figure 4:**
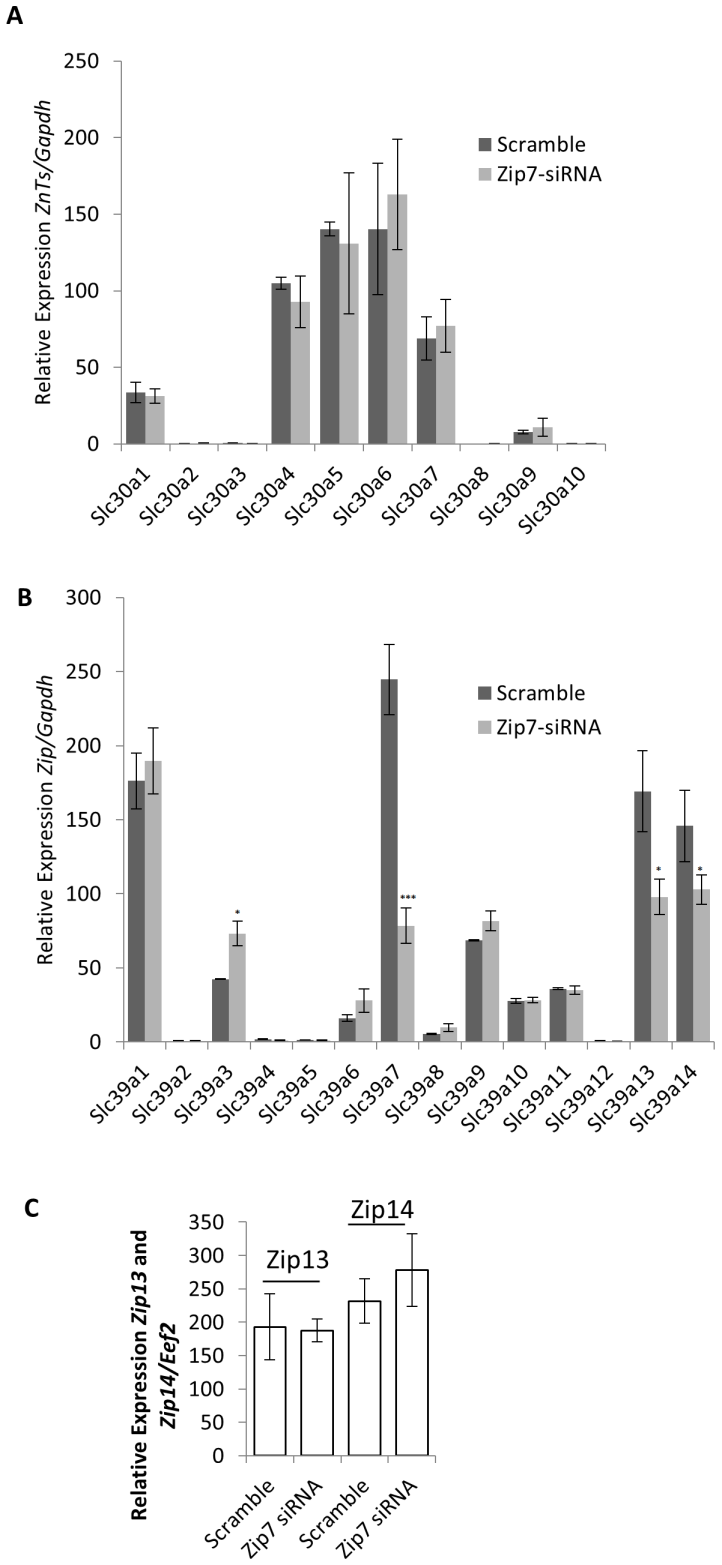
Reduced Zip7 expression has minimal influence on the expression of other zinc transporter genes. A). Relative expression of the *ZnT*s (*Slc30a1-10*) to *Gapdh* in the scramble control versus the siRNA-*Zip7*. B). Relative expression of the *Zip*s (*Slc39a1-14*) to *Gapdh* in the scramble control versus the siRNA-*Zip7*. C and D). Relative expression of *Zip13* and *Zip14*, respectively in the siRNA-*Zip7* cells. Error bars indicated the ± SD from three independent biological samples. *P<0.05, ***P<0.001.

To determine that the attenuation of *Zip7* was not due to differential *Eef2* mRNA expression, qPCR was also performed on *Eef2* normalized to *Gapdh*. No change in the level of *Eef2* in the *Zip7*-siRNA cell lines were observed when normalized to *Gapdh* mRNA (Figure 3C). We also tested the relative expression of *Zip7* in siRNA-*Gapdh* C2C12 cells. There was no change in *Zip7* mRNA expression in the *Gapdh* reduced C2C12 cell lines (Figure 4D).

Since *Zip1* was also highly expressed in C2C12 skeletal muscle cells (Figure 1) we decided to selectively reduce the expression of this transporter with an siRNA-*Zip1* to determine if there were any compensatory changes in *Zip7* expression. C2C12 cells were transfected with the scramble control and siRNA-*Zip1* and endogenous *Zip1* and *Zip7* mRNA was measured. We successfully attenuated endogenous levels of *Zip1* mRNA (approximately 3-fold, p = 0.0025) in the C2C12 cell lines (Figure 3E). No change in endogenous expression of *Zip7* mRNA (p = 0.1040) was observed in the siRNA-*Zip1* cell lines (Figure 3F).

### The attenuation of Zip7 resulted in no change in other zinc transporters

To determine the expression status of the other zinc transporter family members in the presence of the *Zip7* reduced C2C12 cell lines we utilized a custom gene array that contains the primer sequences for the *Slc30a*/*ZnT* (*1–10*) and *Slc39a/Zip* (*1–14*) family members. cDNA from the scramble control and the siRNA-*Zip7* C2C12 cells were assayed to assess for compensatory changes in the other family members due to reduced *Zip7* mRNA. We identified that the reduction of *Zip7* had no effect on the expression of the *Slc30a/ZnT* family members (Figure 4A). In the *Slc39a/Zip* arrays, reduced expression of *Zip7* resulted in a significant attenuation of *Zip7* mRNA as expected. We also observed a small, but significant reduction in the expression of *Zip13* and *Zip14* mRNA (Figure 4B).

To further assess the reduced expression of *Zip13* and *Zip14* in the *Zip7* reduced C2C12 cells we designed primer pairs specific for *Zip13* and *Zip14* to independently test the validity of this observation. We performed qPCR on *Zip13* and *Zip14* expression in the scramble control and siRNA-*Zip7* C2C12 cells. No significant changes in the level of expression for these zinc transporters were observed (Figure 4C).

### Attenuation of Zip7 mRNA in C2C12 cells is associated with changes in several genes implicated in glucose metabolism

We utilized a Mouse Glucose Metabolism RT^2^ Profiler PCR Array (SABiosciences, Qiagen) that contains profiles for the expression of 84 key genes implicated in the regulation of enzymatic pathways of glucose and glycogen metabolism to assess potential pathways that are modulated by *Zip7* (Table S1). We observed that the attenuation of *Zip7* mRNA in C2C12 skeletal muscle cells resulted in changes in several genes implicated in glucose metabolism. These include *Agl* (Amylo-1,6-glucosidase, 4 alpha-glucanotransferase, p = 0.002997), *Dlst* (Dihydrolipoamide S-acetyltransferase, p = 0.035894), *Galm* (Galactose mutarotase, p = 0.001714), *Gbe1* (Glucan-1,4-alpha branching enzyme 1, p = 0.003227), *Idh3g* (Isocitrate dehydrogenase 3 NAD+ gamma, p = 0.015324), *Pck2* (Phosphoenolpyruvate carboxykinase 2, p = 0.002191), *Pgam2* (Phosphoglycerate mutase 2, p = 0.031514), *Pgm2* (Phosphoglucomutase 2, p = 0.027981), *Phkb* (Phosphorylase kinase beta, p = 0.032247), *Pygm* (Muscle glycogen phosphorylase, p = 0.004097), *Tpi1* (Triosephosphate isomerase 1, p = 0.021080) and *Gusb* (Glucuronidase beta, p = 0.013637) (Table 1 and Table S1).

**Table 1:** Fold changes in expression of glucose metabolic genes in the siRNA-Zip7 compared to the scramble control.

PATHWAY: GLUCOSE METABOLISM				
***GLYCOLYSIS***			**T-TEST**	**Fold Up- or Down-Regulation**
**Gene Symbol**	***Description***	**Gene Name**	**p value** *	**siRNA-Zip7/Scramble**
NM_176963	*Galm*	Galactose mutarotase	0.00171	−1.71
NM_010368	*Gusb*	Glucuronidase, beta	0.01363	−1.15
NM_018870	*Pgam2*	Phosphoglycerate mutase 2	0.03151	−1.6
NM_028132	*Pgm2*	Phosphoglucomutase 2	0.02798	−1.36
NM_009415	*Tpi1*	Triosephosphate isomerase 1	0.02108	−1.24
***GLUCONEOGENESIS***			**T-TEST**	**Fold Up- or Down-Regulation**
**Gene Symbol**	**Description**	**Gene Name**	**p value** *	**siRNA-Zip7/Scramble**
NM_028994	*Pck2*	Phosphoenolpyruvate carboxykinase 2 (mitochondrial)	0.00219	1.82
***TCA CYCLE***			**T-TEST**	**Fold Up- or Down-Regulation**
**Gene Symbol**	**Description**	**Gene Name**	**p value** *	**siRNA-Zip7/Scramble**
NM_030225	*Dlst*	Dihydrolipoamide S-succinyltransferase	0.03589	−1.14
NM_008323	*Idh3g*	Isocitrate dehydrogenase 3 (NAD+), gamma	0.01532	−1.31

PATHWAY: GLYCOGEN METABOLISM				
***SYNTHESIS***			**T-TEST**	**Fold Up- or Down-Regulation**
**Gene Symbol**	**Description**	**Gene Name**	**p value** *	**siRNA-Zip7/Scramble**
NM_028803	*Gbe1*	Glucan (1,4-alpha-), branching enzyme 1	0.00322	−1.98
***DEGRADATION***			**T-TEST**	**Fold Up- or Down-Regulation**
**Gene Symbol**	**Description**	**Gene Name**	**p value** *	**siRNA-Zip7/Scramble**
NM_001081326	*Agl*	Amylo-1,6-glucosidase, 4-alpha-glucanotransferase	0.00299	−1.41
NM_011224	*Pygm*	Muscle glycogen phosphorylase	0.00409	−1.75
***REGULATION***			**T-TEST**	**Fold Up- or Down-Regulation**
**Gene Symbol**	**Description**	**Gene Name**	**p value** *	**siRNA-Zip7/Scramble**
NM_199446	*Phkb*	Phosphorylase kinase beta	0.03224	−1.43

* P values <0.05

A Mouse Glucose Metabolism RT2 Profiler PCR Array was utilized to profile the expression of 84 genes involved in the regulation and enzymatic pathways of glucose and glycogen metabolism. Three independent biological samples were utilized and the data was considered statistically significance when the P value was ≤0.05.

**Figure 5:**
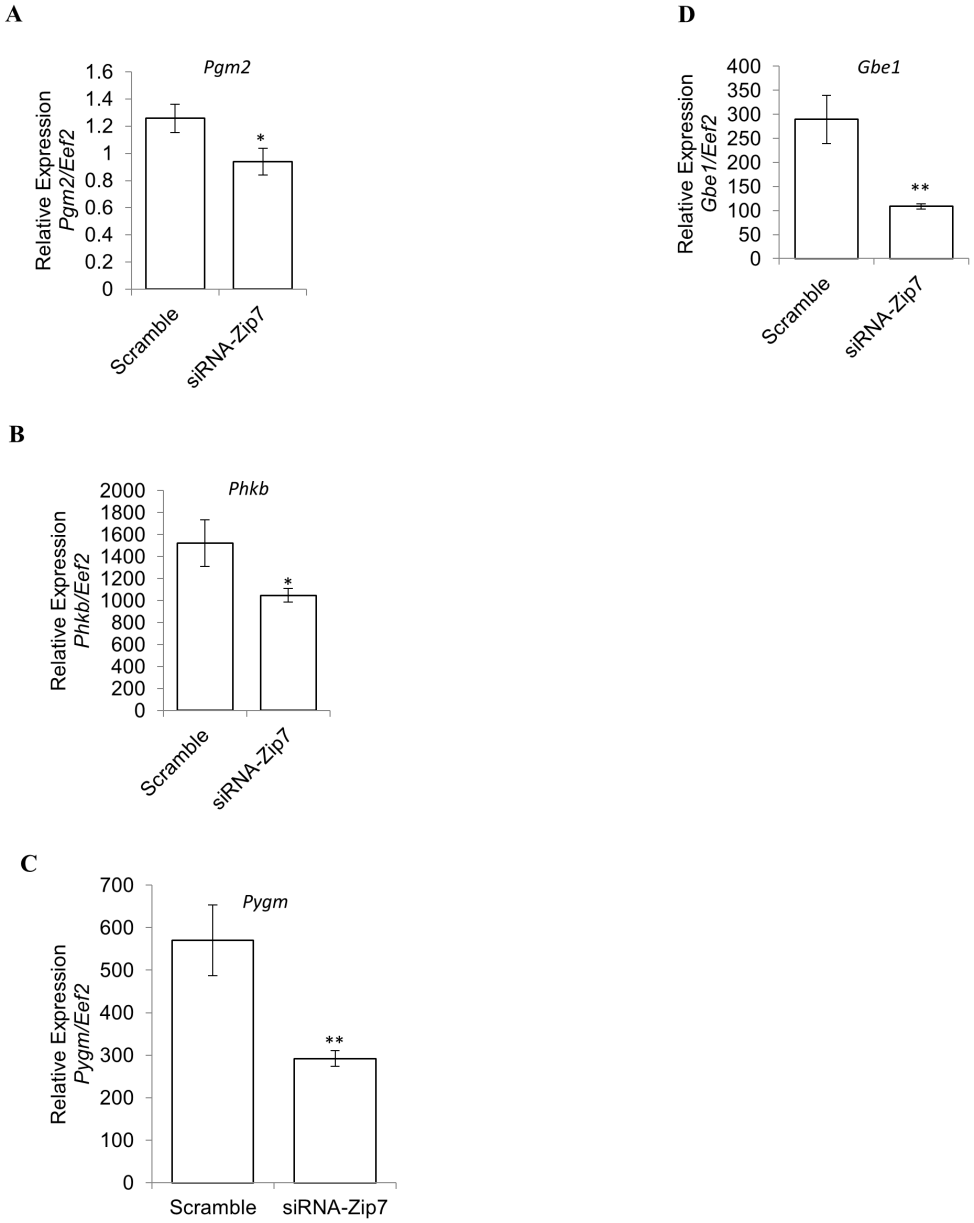
Reduced Zip7 expression alters gene expression of key glucose metabolic genes. A–E). Relative expression of *Pgm2, Phkb, Pygm* and *Geb1* mRNA to *Eef2* in the scramble control and the siRNA-Zip7, respectively. Error bars indicated the ± SD from three independent biological samples. *P<0.05, **P<0.01.

We further validated several of these genes with a focus on glycogen metabolism (*Pgm2, Phkb, Pygm* and *Gbe1*) by designing new primer pairs and performing qPCR on the scramble control versus the siRNA-*Zip7* cDNA. We observed significant downregulation in these genes in concordance with the PCR array data (Figure 5A–D).

**Figure 6:**
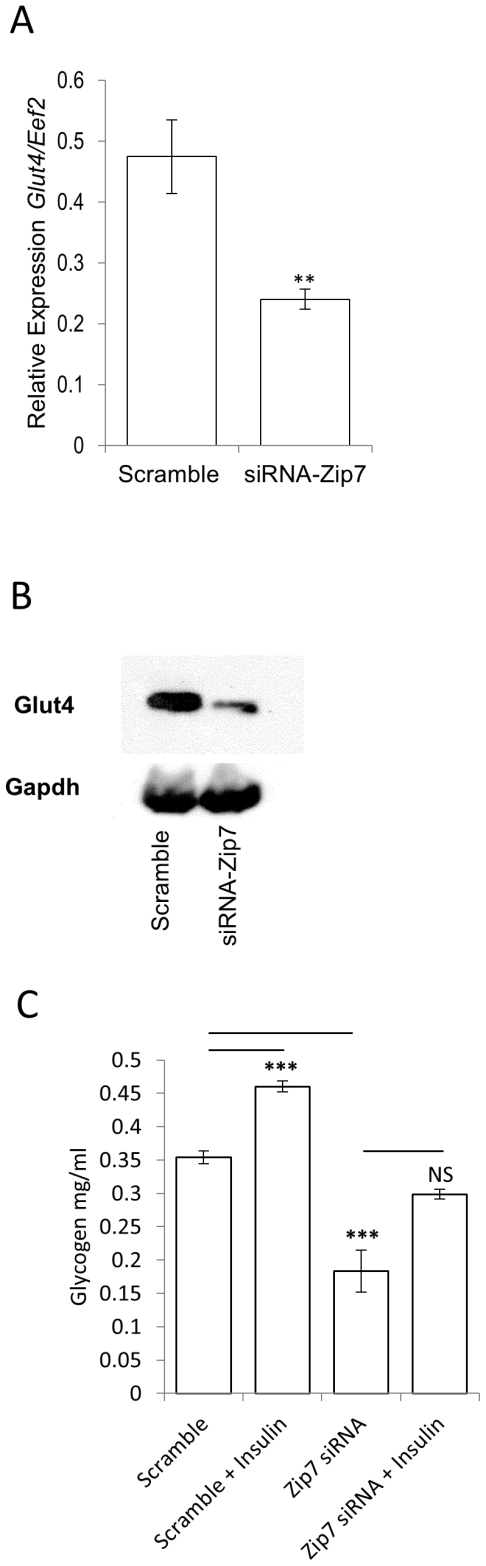
Reduced Zip7 expression reduces the expression of Glut4 and decreases glycogen synthesis. A). Relative expression of *Glut4* mRNA to *Eef2* in the scramble control and siRNA-*Zip7* C2C12 cells. B). Western blot for immunoreactive Glut4 and Gapdh in protein lysates from the scramble control and the siRNA-*Zip7* C2C12 cells. C). Assay for glycogen synthesis in scramble and siRNA-*Zip7* cells treated with 10 nM insulin for 1 hour. D). Error bars indicated the ± SD from three independent biological samples for *Glut4* mRNA, and six independent transient transfections of the siRNA-*Zip7* for the glycogen synthesis. **P<0.01, ***P<0.001.

We speculated that given genes implicated in glycogen metabolism were affected by reduced *Zip7* mRNA levels, that perhaps the glucose transporter, *Glut4* might be downregulated in the siRNA-*Zip7* cells. Glut4 predominately transports glucose across the plasma membrane which is further processed by oxidative (glycolysis) or non-oxidative (glycogenesis) pathways [32]. Accordingly, qPCR was performed for *Glut4* mRNA expression in the scramble control and the siRNA-*Zip7* C2C12 cells. We observed a significant downregulation of *Glut4* in the siRNA-*Zip7* cells (p = 0.0096) (Figure 6A). We also tested for Glut4 immunoreactive protein in the scramble control and siRNA-*Zip7* C2C12 cells. Accordingly we observed a significant reduction in immunoreactive Glut4 in the siRNA-*Zip7* C2C12 cells compared to the scramble control (Figure 6B). Gapdh was used as a protein loading control and showed that similar amounts of total soluble protein were resolved (Figure 6B).

### Reduced Zip7 compromises insulin-induced glycogen synthesis and phosphorylation of AKT in C2C12 skeletal muscle cells

Cellular glucose utilization by Glut4 is responsible for glycogenesis in muscle [33] and with increasing plasma insulin concentration, glycogen synthase is activated by insulin and glycogen synthesis predominates [34]. Moreover, a core component of glycogen synthesis is the insulin-induced phosphorylation of AKT in a process that leads to the activation of glycogen synthase [34]. To test the efficacy of insulin to induce phosphorylation of Akt and thus confirm the robustness of the C2C12 skeletal muscle cell line to respond to insulin, skeletal muscle cells were treated with 10 nM insulin over 60 mins mins and subsequent protein was extracted as described in Material and Methods. We observed that 10 nM of insulin activated pAkt after 5 min followed by a robust phosphorylation of Akt over the 60 min time course (Figure S1), and thus confirmed the validity of skeletal muscle cell line to respond to insulin treatment (Figure S1). Similarly, to test whether maintaining the C2C12 line in the presence of 20 µM ZnSO_4_ (see Material and Methods, Cell culture) affected the phosphorylation status of AKT we treated cells with ZnSO_4_ alone and ZnSO_4_ in the presence of an ionphore, pyrithione (Figure S2). Accordingly, 10 µM of pyrithione in the presence of 20 µM ZnSO_4_ induced a rapid phosphorylation of AKT within 15 minutes and this increased further over 30 and 60 minutes of treatment (Figure S2). We did not observe an increase in AKT phosphorylation in the presence of ZnSO_4_ alone and thus confirmed that maintaining our cell culture system in the presence of 20 µM ZnSO_4_ had no effect on AKT phosphorylation.

Given that *Zip7* modulates core genes implicated in glucose metabolism, we tested whether glycogen synthesis was compromised in the attenuated *Zip7* skeletal muscle cells. We treated the scramble and siRNA*-Zip7* C2C12 cells with 10nM insulin over 60 minutes and performed glycogen synthesis. We observed a significant reduction in glycogen synthesis in the siRNA-*Zip7* when compared to the scramble control (Figure 6C). As expected, we observed a significant induction of glycogen synthesis on exposure to insulin in the scramble control cells, however this effect was blunted in the *Zip7*-siRNA C2C12 (Figure 6C).

**Figure 7:**
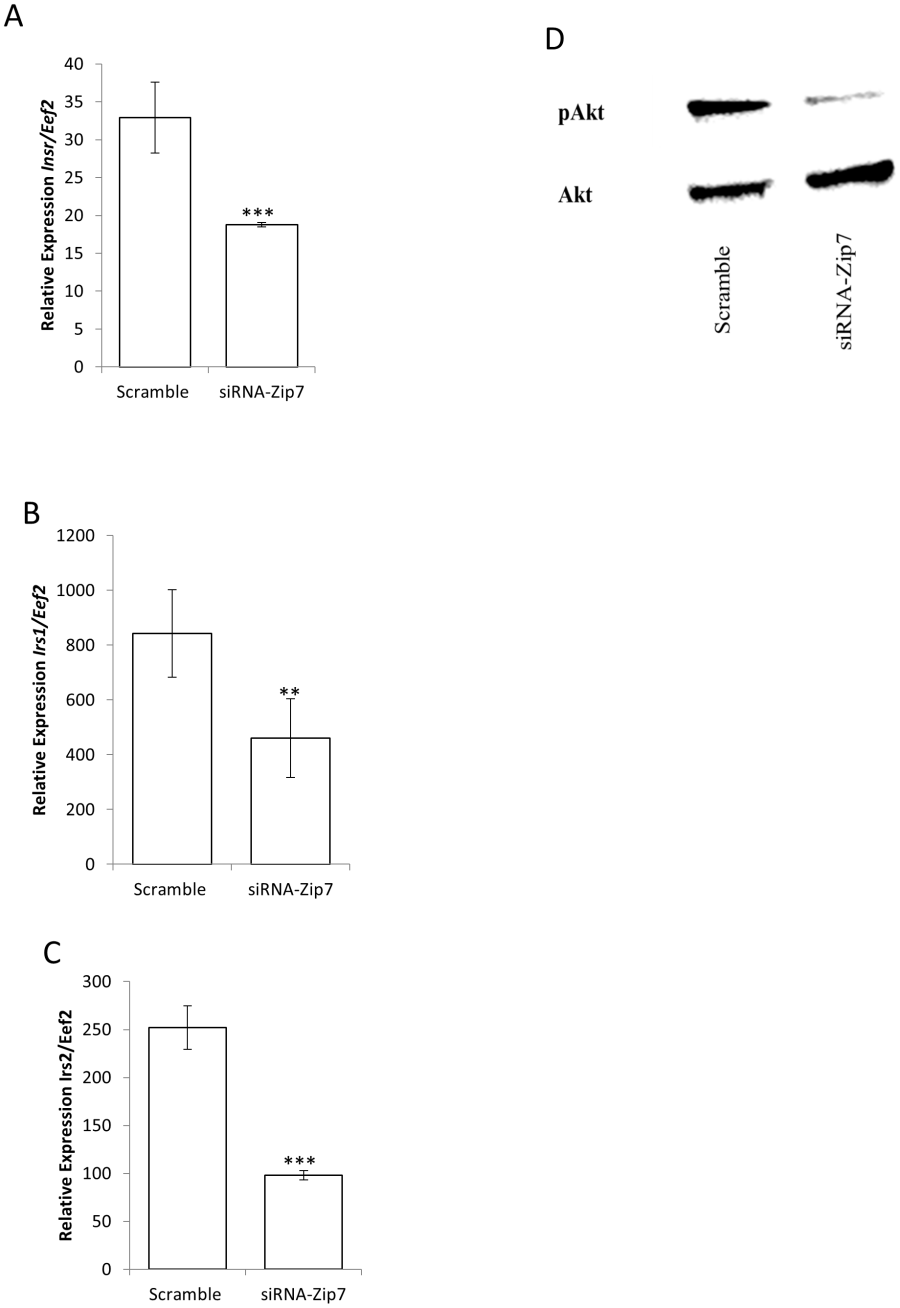
Reduced Zip7 expression reduces the mRNA expression of the insulin receptor (Insr), insulin receptor substrate 1 (Irs1), insulin receptor substrate 2 (Irs2) and the phosphorylation of AKT. A–C). Relative expression of *Insr*, *Isr1* and *Isr2* mRNA to *Eef2* in the scramble control and the siRNA-*Zip7* C2C12 skeletal muscle cells. D. Western blot for immunoreactive pAkt and Akt in protein lysates isolated from scramble control and siRNA-*Zip7* transfected C2C12 skeletal muscle cells. Error bars indicate the ± SD from three independent biological samples for the mRNA analysis of *Inrs, Irs1* and Irs2. **P<0.01, ***P<0.001. Western blot analysis for pAkt and Akt was performed three times on six independent and pooled transient transfections of the scramble control and siRNA-*Zip7.*

To determine a potential mechanism of action for the reduced glycogen synthesis in the presence of reduced Zip7 mRNA we performed qPCR on the insulin receptor (*Insr*) and the most predominant isoforms of the insulin receptor substrate molecules that are expressed in skeletal muscle, insulin receptor substrate 1 (*Irs1*), and insulin receptor substrate 2 (*Irs2*) [32]. These substrates serve as docking molecules for several SH2-containing proteins and the subsequent activation of downstream signaling molecules that result in the activation of AKT, which mediates many of insulin's metabolic effects by modulating gluconeogenesis, protein synthesis and glycogen synthesis [33]. Accordingly, the reduced expression of *Zip7* in the C2C12 skeletal muscle cells resulted in a significant reduction in the expression of the *Insr*, *Irs1* and *Irs2* (Figure 7A–C). In order to confirm that the reduction of these key genes was associated with a reduction in signaling we performed immunoblot analysis on phosphorylated Akt (pAkt). We observed a significant reduction in pAkt in the *Zip7*-siRNA compared to the scramble control (Figure 7D).

### Overexpression of Zip7 in C2C12 cells induces genes associated with glucose metabolism

**Figure 8:**
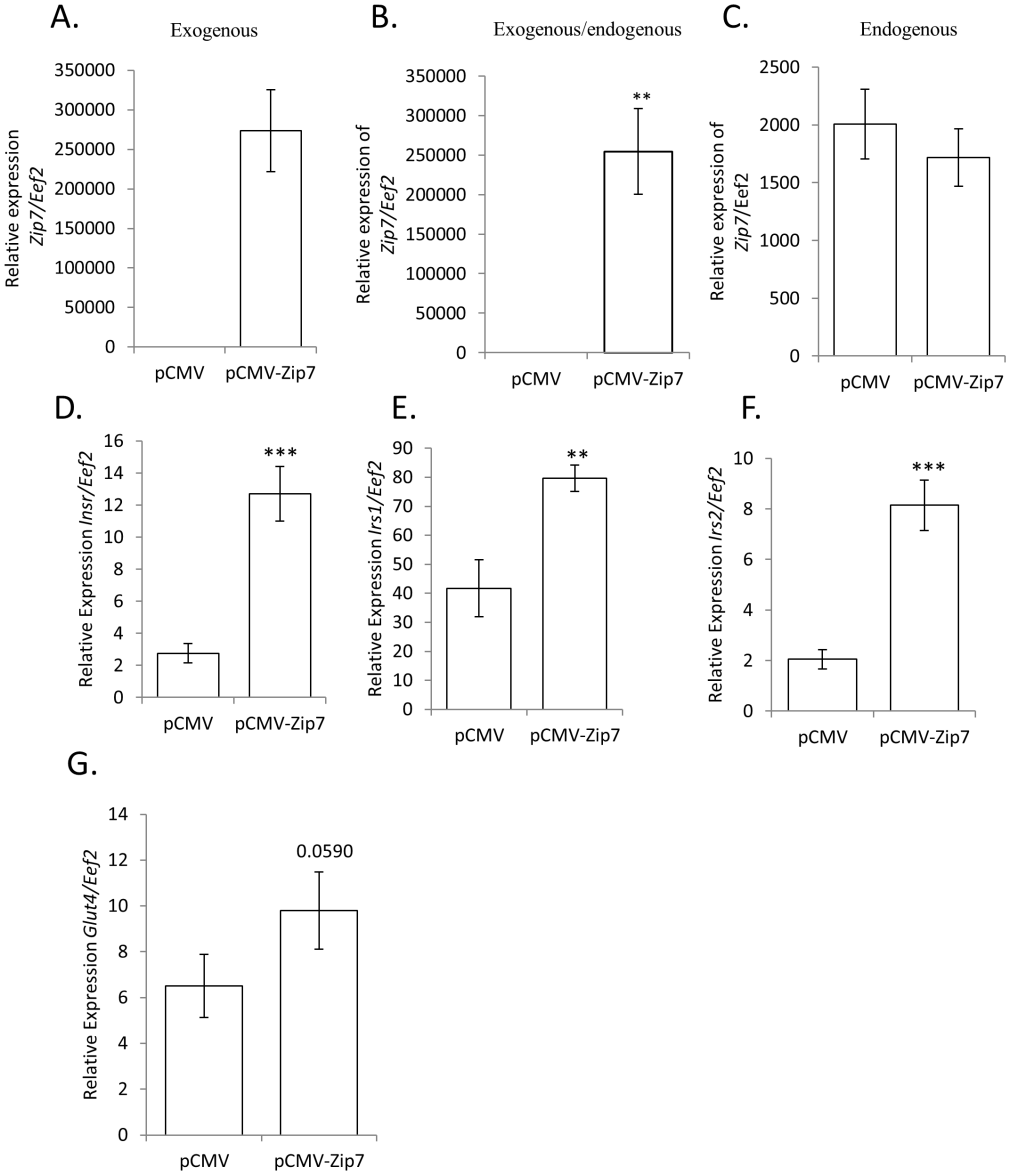
The exogenous overexpression of *Zip7* mRNA induces the expression of the insulin receptor (*Insr*), insulin receptor substrate 1 (*Irs1*), insulin receptor substrate 2 (*Isr2*) and *Glut4* mRNA. A. Relative expression of exogenous *Zip7* mRNA. B. Relative expression of endogenous and exogenous Zip7 *mRNA*. C. Relative expression of endogenous expression of *Zip7* mRNA. D-F. Relative expression of *Insr, Irs1* and *Irs2* mRNA. G. Relative expression of *Glut4* mRNA (not significant P = 0.0590). Error bars indicate the ± SD from two independent biological samples for the mRNA analysis that consisted of at least three independent transfections. **P<0.01, ***P<0.001.

We observed that reduced *Zip7* mRNA in C2C12 skeletal muscle cells was associated with changes in genes implicated in glucose metabolism. For example, a significant reduction in the expression of *Pgm2, Phkb, Pygm, Gbe1, Glut4*, *Insr, Irs1* and *Irs2* was observed in the *Zip7*-siRNA C2C12 cells compared to the scramble control (see Figures 5, 6, 7). Accordingly, to determine if by overexpressing *Zip7* we could observe the converse effect on gene expression, we transiently transfected an overexpression *Zip7* plasmid (pCMV-Zip7) into C2C12 skeletal muscle cells and after 72 hours collected RNA for subsequent qPCR analysis. We observed a significant induction in the expression of exogenous *Zip7* in the pCMV-Zip7 expressing C2C12 cells compared to the pCMV control (Figure 8A and B). To confirm that the major *Zip7* mRNA transcript observed was from the overexpression of the pCMV-*Zip7* plasmid we performed PCR using primers that were specific for the endogenous form of *Zip7* mRNA. We observed that Zip7 mRNA was expressed at relatively much lower levels in both the pCMV and pCMV-Zip7 transfected cells (Figure 8C) confirming that the major *Zip7* transcript resulted from the overexpression system.

Moreover, we found that the overexpression of Zip7 mRNA induced the expression of the insulin receptor (*Insr*); insulin receptor substrate 1 (*Isr1*) and insulin receptor substrate 2 (*Isr2*) (see Figure 8D–F). This was in contrast to Figure 7 where a reduction in the expression of *Zip7* mRNA resulted in reduced expression of *Insr, Isr1* and *Isr2*. We also observed an increase in *Glut4* mRNA in the pCMV-Zip7 overexpression system, however this result did not attain significance (p = 0.0590). Similarly, Glut4 protein levels were not significantly changed in the pCMV-Zip7 overexpression system when compared to the pCMV control (data not shown).

## Discussion

Intracellular zinc homeostasis is largely regulated by two families of zinc transporters (ZnTs and ZIPs) that traffic zinc across biological membranes [35], [36]. Dysregulation of zinc signaling leads to a number of disease states including cancer [19], [37], autoimmune disease [38], [39], cardiovascular disease [40], [41] and diabetes [42]–[45]. Of this family, ZIP7 is important in maintaining physiological and cellular zinc homeostasis through its ability to initiate the ‘zinc wave’ and provide cytosolic zinc ions that are involved in cellular signaling processes. Although many zinc transporters respond to fluctuating zinc levels and alter their subcellular localization, ZIP7 is an exception and is restricted constitutively to the membrane of the Golgi apparatus and/or the endoplasmic reticulum [15]–[17]. Furthermore, *Zip7* gene expression and intracellular location are not altered in response to changes in intracellular zinc status [3]. Studies in breast cancer cells have elucidated a role for this transporter in cell signaling events [8], [20]; however, the role of ZIP7 with respect to the control of the genetic programs associated with carbohydrate metabolism in skeletal muscle has not been addressed. Here we provide the first evidence for a metabolic role for *Zip7* in modulating glycaemic control in skeletal muscle and provide support for further studies in processes associated with insulin resistance in this tissue.

*Zip7* mRNA is highly expressed in differentiated C2C12 cells and mouse quadriceps. Although *Slc39a1* was also highly expressed in C2C12 skeletal muscle cells, homozygous knockout of *Slc39a1* in mice produces no phenotype when dietary zinc intake is normal [46] suggesting compensatory actions from other family members. To explore compensatory mechanisms from other zinc transporters we performed qPCR on all of the family members in the scramble control and the siRNA-*Zip7* C2C12 skeletal muscle cells. We did not observe major changes in expression of the other members of the zinc transporters which suggest that the attenuation of *Zip7* has no other effect on these genes. Of the zinc transporters, it should be emphasized that, in addition to ZIP13, [47] ZIP7 is the only other zinc transporter localized to the Golgi apparatus and not the plasma membrane [15] and compensation is therefore unlikely. Given that ZIP7 is localized exclusively on the Golgi apparatus or the ER [17], and the fact that no compensatory changes in the other transporters were observed in the reduced *Zip7* C2C12 cells suggests that this transporter may be unique in its specialized function in transporting zinc from the ER or Golgi into the cytosol [19].

In contrast to the *Zip* expression profile in C2C12 cells, we also observed moderate levels of expression for all of the *Zip* transporters (except for *Zip5*) in mouse quadriceps. *Zip7* mRNA was more highly expressed in C2C12 cells (approximately 15-fold) when compared to the expression found in quadriceps. It should be noted that quadriceps contain a mix of muscle fibre-types (oxidative type 1 and glycolytic type II) [48]. Similar studies on other muscle fibre types; soleus (type I), plantaris (type II) and anterior tibialis (type II) also demonstrated differences in the level of expression for the orphan nuclear receptor, *Coup-tfII* in these tissues in comparison to C2C12 cells [49]. Moreover, studies on protein arginine methyltransferase 3, 4 and 5 (PRMT3–5) in mouse skeletal muscle tissue and C2C12 cells found high expression of PRMT3–5 in gastrocnemius in comparison to only high expression of PRMT4 (with no or minimal expression of PRMT3 and 5, respectively) [50]. Although these relative expression discrepancies exist between *in vitro* and *in vivo* model systems, the C2C12 cell culture model is a well-established and validated system to study the effects of metabolic processes [21], [51], [52]. For example, data derived from this *in vitro* model with liver X receptor (LXR) and peroxisome proliferating activated receptor (PPAR) agonists and their role in metabolism (e.g. energy expenditure, running endurance, lipid metabolism and cholesterol efflux) has been validated and reproduced in mice [51]–[55].

Our study revealed that subsets of genes involved in glucose metabolism (*Agl, Dlst, Galm, Gbe1*, *Idh3g, Pck2, Pgam2, Pgm2, Phkb, Pygm, Tpi1, Gusb* and *Glut4*) are altered when *Zip7* expression was reduced. This is further highlighted by the fact that related genes in similar and other pathways (see Table S1) were refractory to the attenuation of *Zip7* expression. These data are noteworthy for several reasons. For example, in skeletal muscle, GLUT4 predominately transports glucose across the plasma membrane which is further processed by oxidative (glycolysis) or non-oxidative (glycogenesis) pathways [32]. Thus, the decline in Glut4 protein in the *Zip7*-reduced C2C12 cells would suggest a reduction in glucose transport and subsequent genes associated with oxidative and non-oxidative pathways. Similarly, this is supported by the observation that several genes implicated in glycolysis (*Galm, Gusb, Pgam2, Pgm2* and *Tpi1*) and glycogen synthesis (*Gbe1, Agl, Pgm2, Pygm* and *Phkg2*) were reduced in the *Zip7* attenuated cells. In skeletal muscle, these genes play critical roles in the oxidative and non-oxidative pathways, respectively. This is further supported by the reduction in the mRNA of the insulin receptor (*Insr*), and the insulin receptor substrates 1 and 2 *(Isr1* and *Isr2*) and the subsequent reduction of basal and insulin-mediated glycogen storage in C2C12 myotubes when *Zip7* expression was attenuated (see Figure 6).

Skeletal muscle is particularly important in maintaining glucose homeostasis because approximately 70-90% of whole body insulin-mediated induction of glucose uptake occurs in muscle where it is incorporated into glycogen for storage [56]. Moreover, in insulin-resistant states, insulin-induced glucose uptake and glycogen synthesis is markedly reduced in skeletal muscle [32], [57]. Accordingly, in association with a reduction in genes involved in glycogen metabolism and the fact that there was a reduction in glycogen synthesis, we also observed a significant decrease in the phosphorylation status of AKT in the reduced *Zip7* expressing C2C12 cells. This is consistent with recent studies where a siRNA targeting *ZIP7* significantly decreased zinc-induced pAKT after 5 minutes of 20 µM zinc treatment in MCF-7 tamoxifen-resistant breast cancer cells [8]. Moreover, in a recent study in a *Zip9* gene knockout chicken DT40 cell model, the levels of phosphorylation of Akt and Erk were significantly reduced [58]. Given the role of Zip7 in facilitating zinc flux into the cytosol [8], and the fact that previous studies have shown that zinc can activate pAKT [8], [59], it will be important to determine whether Zip7 in skeletal muscle plays a similar role in mediating zinc flux and signaling events that lead to phosphorylation of AKT and the mobilization of glucose transporters.

Zinc is a well-known inhibitor of protein tyrosine phosphatases (PTPs) [60] with a reported inhibition constant in the nanomolar range [61]. Zinc inhibits PTP1B, a cytoplasmic phosphatase that interacts with the insulin receptor and catalyzes its dephosphorylation resulting in the attenuation of insulin signaling [62]. Based on these results, and the fact that the insulin signaling pathway depends on the status of tyrosine phosphatases and the release of zinc into the cytosol, we hypothesize that reduced expression of *Zip7* could lead to a reduction in the cytosolic zinc pool that is available for cellular signaling. For example, in the testes of diabetic mice treated with the zinc chelator, TPEN, a significant down-regulation of Akt-mediated glucose metabolism signaling was observed that was reflected by reduced phosphorylation of Akt and Gsk-3β [63]. Moreover, treatment of 3T3-L1 adipocytes with ZnCl_2_ increased tyrosine phosphorylation of the insulin receptor beta subunit and enhanced the transport of glucose in the absence of insulin through the PI3-kinase/Akt pathway [53]. Furthermore, in myocytes isolated from the femoral muscle of mice with a ZnT7 knock-out (these mice display low zinc status) there was reduced insulin signaling pathway activity and these mice were insulin resistant. This was also congruent with a reduction in the mRNA expression of *Insr*, *Irs2* and *Akt* [3].

Based on the observations that Zip7 plays a crucial role in facilitating cytosolic zinc flux [8], and given the role of zinc as a second messenger that activates pathways associated with cellular signaling, these studies now show a new role for *Zip7* in regulating the critical gene programs involved in glucose uptake and glycogen storage in skeletal muscle. In particular, the mRNA down-regulation of *Insr*, *Irs1* and *Irs2*, in association with reduced phosphorylation of Akt and reduced Glut4 expression, suggests that Zip7 activity may be amenable to manipulation as a novel approach for the treatment of insulin resistance in skeletal muscle.

## Supporting Information

Figure S1 A. Western blot analysis for insulin-induced phosphorylation of AKT in C2C12 skeletal muscle cells. C2C12 skeletal muscle cells were differentiated in 2% horse serum for 3 days and then treated in the absence or presence of 10 nM of insulin for 60 minutes. Total cellular protein was collected and the presence for immunoreactive pAkt and Akt was assessed. This immunoblot is a representation of three independent biologically insulin-treated C2C12 cell preparations. B. Average densitometry quantification of pAkt/Akt. pAkt quantified by densitometry on immunoblots from three independent experiments normalized to total Akt and displayed as the mean ± SD with significant (**P≤0.01,*** P≤0.001) changes over time 0.
(TIF)

Figure S2 Western blot analysis for zinc induced phosphorylation of AKT in the absence and presence of 10 µM pyrithione in C2C12 skeletal muscle cells. C2C12 skeletal muscle cells were differentiated in 2% horse serum for 3 days and then treated in the presence (+) or absence (-) of 10 µM of pyrithione over 60 minutes. Total cellular protein was extracted and the presence for immunoreactive pAKT and AKT was performed by western blot analysis. This immunoblot represents at least three independent biological replicates.
(TIF)

Table S1 Fold changes in expression of glucose metabolic genes in the siRNA-*Zip7* compared to the scramble control.
(DOC)

Table S2 Primer sequences for the amplification of target genes.
(DOC)
