# Supplementary figures and images for: The Zinc Transporter, Slc39a7 (Zip7) Is Implicated in Glycaemic Control in Skeletal Muscle Cells
Source: PLoS One. 2013 Nov 12;8(11):e79316. doi: 10.1371/journal.pone.0079316 (PMC3827150; doi:10.1371/journal.pone.0079316)

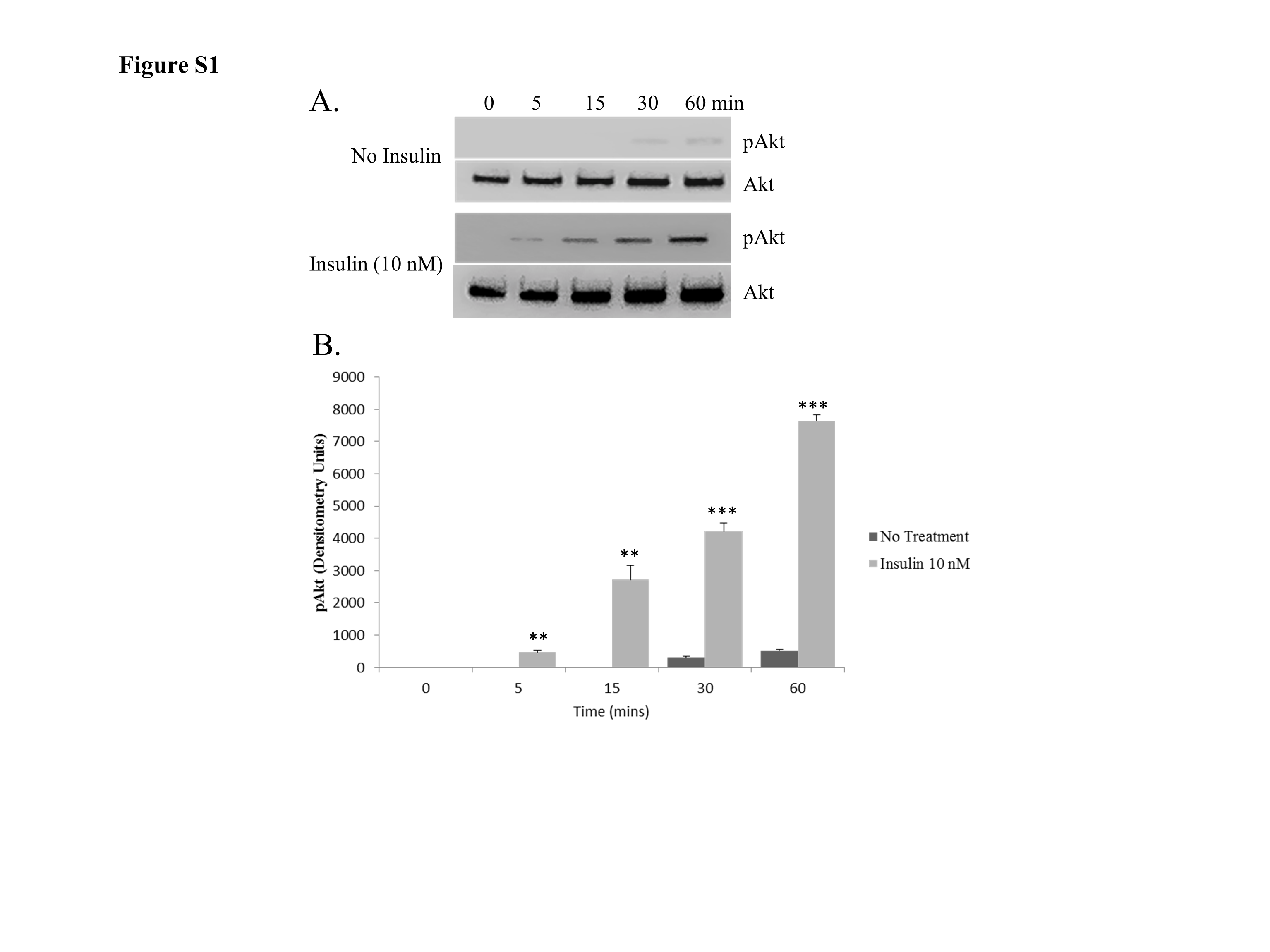

Supplement: Figure S1 — A. Western blot analysis for insulin-induced phosphorylation of AKT in C2C12 skeletal muscle cells. C2C12 skeletal muscle cells were differentiated in 2% horse serum for 3 days and then treated in the absence or presence of 10 nM of insulin for 60 minutes. Total cellular protein was collected and the presence for immunoreactive pAkt and Akt was assessed. This immunoblot is a representation of three independent biologically insulin-treated C2C12 cell preparations. B. Average densitometry quantification of pAkt/Akt. pAkt quantified by densitometry on immunoblots from three independent experiments normalized to total Akt and displayed as the mean ± SD with significant (**P≤0.01,*** P≤0.001) changes over time 0. (TIF) [file pone.0079316.s001.tif]

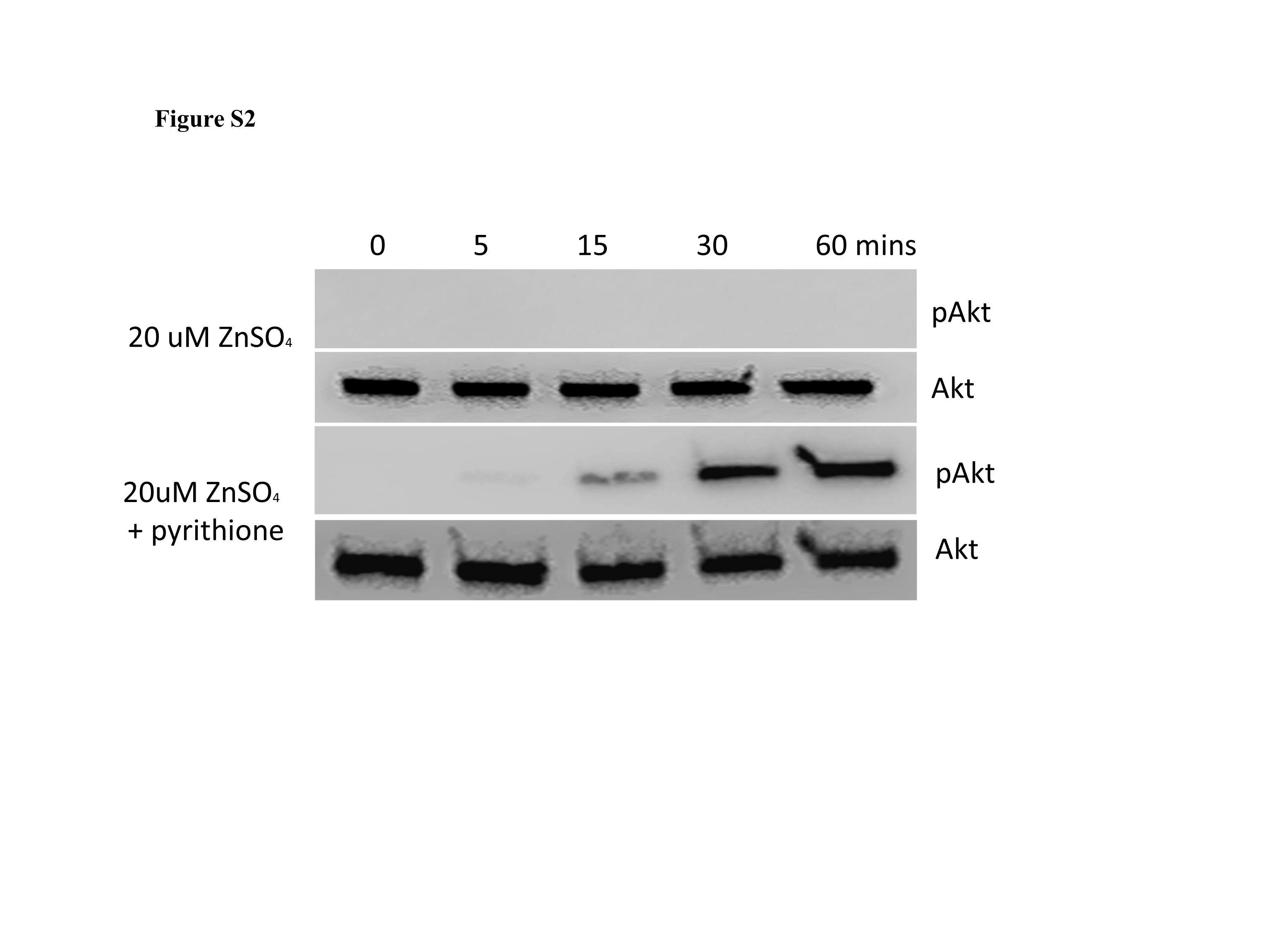

Supplement: Figure S2 — Western blot analysis for zinc induced phosphorylation of AKT in the absence and presence of 10 µM pyrithione in C2C12 skeletal muscle cells. C2C12 skeletal muscle cells were differentiated in 2% horse serum for 3 days and then treated in the presence (+) or absence (-) of 10 µM of pyrithione over 60 minutes. Total cellular protein was extracted and the presence for immunoreactive pAKT and AKT was performed by western blot analysis. This immunoblot represents at least three independent biological replicates. (TIF) [file pone.0079316.s002.tif]
