# Supplementary material for: The Zinc Transporter, Slc39a7 (Zip7) Is Implicated in Glycaemic Control in Skeletal Muscle Cells
Source: PLoS One. 2013 Nov 12;8(11):e79316. doi: 10.1371/journal.pone.0079316 (PMC3827150; doi:10.1371/journal.pone.0079316)
Supplement: Table S1 — Fold changes in expression of glucose metabolic genes in the siRNA-Zip7 compared to the scramble control. (DOC) [file pone.0079316.s003.doc]

Table S1: Fold changes in expression of glucose metabolic genes in the siRNA-*Zip7* compared

to the scramble control

| **PATHWAY: GLUCOSE METABOLISM** | | | | |
| --- | --- | --- | --- | --- |
| ***GLYCOLYSIS*** | | | **T-TEST** | **Fold Up- or Down-Regulation** |
| **Gene Symbol** | **Description** | **Gene Name** | **p value^*^** | **siRNA-Zip7 /Scramble** |
| NM_007438 | *Aldoa* | Aldolase A, fructose-bisphosphate | 0.08947 | -1.25 |
| NM_144903 | *Aldob* | Aldolase B, fructose-bisphosphate | 0.33316 | -1.32 |
| NM_009657 | *Aldoc* | Aldolase C, fructose-bisphosphate | 0.87102 | 1.04 |
| NM_007563 | *Bpgm* | 2,3-bisphosphoglycerate mutase | 0.30321 | -1.24 |
| NM_023119 | *Eno1* | Enolase 1, alpha non-neuron | 0.52365 | -1.08 |
| NM_013509 | *Eno2* | Enolase 2, gamma neuronal | 0.91352 | 1.06 |
| NM_007933 | *Eno3* | Enolase 3, beta muscle | 0.21283 | -1.22 |
| NM_176963 | *Galm* | Galactose mutarotase | **0.00171** | -1.71 |
| NM_008085 | *Gapdhs* | Glyceraldehyde-3-phosphate dehydrogenase, spermatogenic | 0.88531 | 1 |
| NM_010292 | *Gck* | Glucokinase | 0.37497 | 4.39 |
| NM_008155 | *Gpi1* | Glucose phosphate isomerase 1 | 0.51587 | -1.05 |
| NM_010368 | *Gusb* | Glucuronidase, beta | **0.01363** | -1.15 |
| NM_013820 | *Hk2* | Hexokinase 2 | 0.18738 | -1.1 |
| NM_001033245 | *Hk3* | Hexokinase 3 | 0.26111 | -1.64 |
| NM_008826 | *Pfkl* | Phosphofructokinase, liver, B-type | 0.62272 | -1.07 |
| NM_018870 | *Pgam2* | Phosphoglycerate mutase 2 | **0.03151** | -1.6 |
| NM_008828 | *Pgk1* | Phosphoglycerate kinase 1 | 0.06880 | -1.26 |
| NM_031190 | *Pgk2* | Phosphoglycerate kinase 2 | 0.16190 | -1.25 |
| NM_025700 | *Pgm1* | Phosphoglucomutase 1 | 0.88119 | 1.02 |
| NM_028132 | *Pgm2* | Phosphoglucomutase 2 | **0.02798** | -1.36 |
| NM_028352 | *Pgm3* | Phosphoglucomutase 3 | 0.19209 | 1.32 |
| NM_013631 | *Pklr* | Pyruvate kinase liver and red blood cell | 0.36316 | 1.69 |
| NM_009415 | *Tpi1* | Triosephosphate isomerase 1 | **0.02108** | -1.24 |
| ***GLUCONEOGENESIS*** | | | **T-TEST** | **Fold Up- or Down-Regulation** |
| **Gene Symbol** | **Description** | **Gene Name** | **p value^*^** | **siRNA-Zip7 /Scramble** |
| NM_019395 | *Fbp1* | Fructose bisphosphatase 1 | 0.27588 | 1.32 |
| NM_007994 | *Fbp2* | Fructose bisphosphatase 2 | 0.33273 | 1.34 |
| NM_008061 | *G6pc* | Glucose-6-phosphatase, catalytic | 0.08661 | -1.39 |
| NM_175935 | *G6pc3* | Glucose 6 phosphatase, catalytic, 3 | 0.34489 | -1.09 |
| NM_011044 | *Pck1* | Phosphoenolpyruvate carboxykinase 1, cytosolic | 0.97344 | -1.03 |
| NM_028994 | *Pck2* | Phosphoenolpyruvate carboxykinase 2 (mitochondrial) | **0.00219** | 1.82 |
| NM_008797 | *Pcx* | Pyruvate carboxylase | 0.39140 | 1.12 |
| ***REGULATION*** | | | **T-TEST** | **Fold Up- or Down-Regulation** |
| **Gene Symbol** | **Description** | **Gene Name** | **p value^*^** | **siRNA-Zip7 /Scramble** |
| NM_001024606 | *Pdp2* | Pyruvate dehyrogenase phosphatase catalytic subunit 2 | 0.91575 | -1.01 |
| NM_198308 | *Pdpr* | Pyruvate dehydrogenase phosphatase regulatory subunit | 0.84735 | 1.06 |
| NM_172665 | *Pdk1* | Pyruvate dehydrogenase kinase, isoenzyme 1 | 0.23327 | -1.2 |
| NM_133667 | *Pdk2* | Pyruvate dehydrogenase kinase, isoenzyme 2 | 0.91969 | -1.04 |
| NM_145630 | *Pdk3* | Pyruvate dehydrogenase kinase, isoenzyme 3 | 0.82933 | 1 |
| NM_013743 | *Pdk4* | Pyruvate dehydrogenase kinase, isoenzyme 4 | 0.97897 | 1.03 |
| ***TCA CYCLE*** | | | **T-TEST** | **Fold Up- or Down-Regulation** |
| **Gene Symbol** | **Description** | **Gene Name** | **p value^*^** | **siRNA-Zip7 /Scramble** |
| NM_134037 | *Acly* | ATP citrate lyase | 0.25187 | 1.11 |
| NM_007386 | *Aco1* | Aconitase 1 | 0.55393 | -1.03 |
| NM_080633 | *Aco2* | Aconitase 2, mitochondrial | 0.22127 | 1.14 |
| NM_026444 | *Cs* | Citrate synthase | 0.74872 | -1.03 |
| NM_145614 | *Dlat* | Dihydrolipoamide S-acetyltransferase | 0.27429 | -1.08 |
| NM_007861 | *Dld* | Dihydrolipoamide dehydrogenase | 0.97111 | -1 |
| NM_030225 | *Dlst* | Dihydrolipoamide S-succinyltransferase | **0.03589** | -1.14 |
| NM_010209 | *Fh1* | Fumarate hydratase 1 | 0.15676 | -1.17 |
| NM_010497 | *Idh1* | Isocitrate dehydrogenase 1 (NADP+), soluble | 0.27740 | 1.23 |
| NM_173011 | *Idh2* | Isocitrate dehydrogenase 2 (NADP+), mitochondrial | 0.17079 | -1.2 |
| NM_029573 | *Idh3a* | Isocitrate dehydrogenase 3 (NAD+) alpha | 0.59762 | 1.15 |
| NM_130884 | *Idh3b* | Isocitrate dehydrogenase 3 (NAD+) beta | 0.92849 | 1.01 |
| NM_008323 | *Idh3g* | Isocitrate dehydrogenase 3 (NAD+), gamma | **0.01532** | -1.31 |
| NM_008618 | *Mdh1* | Malate dehydrogenase 1, NAD (soluble) | 0.06742 | -1.26 |
| NM_029696 | *Mdh1b* | Malate dehydrogenase 1B, NAD (soluble) | 0.71596 | 1.31 |
| NM_008617 | *Mdh2* | Malate dehydrogenase 2, NAD (mitochondrial) | 0.25732 | -1.11 |
| NM_010956 | *Ogdh* | Oxoglutarate dehydrogenase (lipoamide) | 0.61829 | -1.08 |
| NM_008797 | *Pcx* | Pyruvate carboxylase | 0.39140 | 1.12 |
| NM_008810 | *Pdha1* | Pyruvate dehydrogenase E1 alpha 1 | 0.51163 | -1.06 |
| NM_024221 | *Pdhb* | Pyruvate dehydrogenase (lipoamide) beta | 0.13041 | -1.13 |
| NM_023281 | *Sdha* | Succinate dehydrogenase complex, subunit A, flavoprotein (Fp) | 0.09856 | -1.2 |
| NM_023374 | *Sdhb* | Succinate dehydrogenase complex, subunit B, iron sulfur (Ip) | 0.06095 | -1.17 |
| NM_025321 | *Sdhc* | Succinate dehydrogenase complex, subunit C, integral membrane protein | 0.08024 | -1.15 |
| NM_025848 | *Sdhd* | Succinate dehydrogenase complex, subunit D, integral membrane protein | 0.57031 | -1.03 |
| NM_011506 | *Sucla2* | Succinate-Coenzyme A ligase, ADP-forming, beta subunit | 0.33036 | -1.1 |
| NM_019879 | *Suclg1* | Succinate-CoA ligase, GDP-forming, alpha subunit | 0.80117 | -1.06 |
| NM_011507 | *Suclg2* | Succinate-Coenzyme A ligase, GDP-forming, beta subunit | 0.09417 | -1.15 |
| ***PENTOSE PHOSPHATE PATHWAY*** | | | **T-TEST** | **Fold Up- or Down-Regulation** |
| **Gene Symbol** | **Description** | **Gene Name** | **p value^*^** | **siRNA-Zip7 /Scramble** |
| NM_008062 | *G6pdx* | Glucose-6-phosphate dehydrogenase X-linked | 0.14298 | 1.29 |
| NM_173371 | *H6pd* | Hexose-6-phosphate dehydrogenase (glucose 1-dehydrogenase) | 0.33551 | 1.18 |
| NM_021463 | *Prps1* | Phosphoribosyl pyrophosphate synthetase 1 | 0.20574 | -1.17 |
| NM_029294 | *Prps1l1* | Phosphoribosyl pyrophosphate synthetase 1-like 1 | 0.16219 | 2.03 |
| NM_026662 | *Prps2* | Phosphoribosyl pyrophosphate synthetase 2 | 0.57748 | 1.05 |
| NM_153196 | *Rbks* | Ribokinase | 0.40156 | 1.2 |
| NM_025683 | *Rpe* | Ribulose-5-phosphate-3-epimerase | 0.25434 | -1.17 |
| NM_009075 | *Rpia* | Ribose 5-phosphate isomerase A | 0.08597 | 1.14 |
| NM_011528 | *Taldo1* | Transaldolase 1 | 0.17667 | -1.17 |
| NM_009388 | *Tkt* | Transketolase | 0.37086 | 1.14 |
| **PATHWAY: GLYCOGEN METABOLISM** | | | | |
| ***SYNTHESIS*** | | | **T-TEST** | **Fold Up- or Down-Regulation** |
| **Gene Symbol** | **Description** | **Gene Name** | **p value^*^** | **siRNA-Zip7 /Scramble** |
| NM_028803 | *Gbe1* | Glucan (1,4-alpha-), branching enzyme 1 | **0.00322** | -1.98 |
| NM_030678 | *Gys1* | Glycogen synthase 1, muscle | 0.08607 | -1.41 |
| NM_145572 | *Gys2* | Glycogen synthase 2 | N/A | -1.27 |
| NM_139297 | *Ugp2* | UDP-glucose pyrophosphorylase 2 | 0.12831 | -1.23 |
| ***DEGRADATION*** | | | **T-TEST** | **Fold Up- or Down-Regulation** |
| **Gene Symbol** | **Description** | **Gene Name** | **p value^*^** | **siRNA-Zip7 /Scramble** |
| NM_001081326 | *Agl* | Amylo-1,6-glucosidase, 4-alpha-glucanotransferase | **0.00299** | -1.41 |
| NM_025700 | *Pgm1* | Phosphoglucomutase 1 | 0.88119 | 1.02 |
| NM_028132 | *Pgm2* | Phosphoglucomutase 2 | **0.02798** | -1.36 |
| NM_028352 | *Pgm3* | Phosphoglucomutase 3 | 0.19209 | 1.32 |
| NM_133198 | *Pygl* | Liver glycogen phosphorylase | 0.95172 | 1.23 |
| NM_011224 | *Pygm* | Muscle glycogen phosphorylase | **0.00409** | -1.75 |
| ***REGULATION*** | | | **T-TEST** | **Fold Up- or Down-Regulation** |
| **Gene Symbol** | **Description** | **Gene Name** | **p value^*^** | **siRNA-Zip7 /Scramble** |
| NM_001031667 | *Gsk3a* | Glycogen synthase kinase 3 alpha | 0.69903 | 1.08 |
| NM_019827 | *Gsk3b* | Glycogen synthase kinase 3 beta | 0.17686 | -1.12 |
| NM_173021 | *Phka1* | Phosphorylase kinase alpha 1 | 0.65851 | 1.11 |
| NM_199446 | *Phkb* | Phosphorylase kinase beta | **0.03224** | -1.43 |
| NM_011079 | *Phkg1* | Phosphorylase kinase gamma 1 | 0.45207 | 1.51 |
| NM_026888 | *Phkg2* | Phosphorylase kinase, gamma 2 (testis) | 0.18410 | 1.22 |

^*^ P values in bold font are significant.
