# Supplementary material for: The Zinc Transporter, Slc39a7 (Zip7) Is Implicated in Glycaemic Control in Skeletal Muscle Cells
Source: PLoS One. 2013 Nov 12;8(11):e79316. doi: 10.1371/journal.pone.0079316 (PMC3827150; doi:10.1371/journal.pone.0079316)
Supplement: Table S2 — Primer sequences for the amplification of target genes. (DOC) [file pone.0079316.s004.doc]

**Table S2:** Primer sequences for the amplification of target genes.

| Myogenin | 5’-CCTTAAAGCAGAGAGCATCC-3’ | 5’-GGAATTCGAGGCATAATATGA-3’ |
| --- | --- | --- |
| *Tnni1* | 5’-GCCTATGCGCACACCTTTG-3’ | 5’-CGGGTACCATAAGCCCACACT-3’ |
| *Tnni2* | 5’- AAATGTTCGAGTCTGAGTCCTAACTG-3’ | 5’-GCCAAGTACTCCCAGACTGGAT-3’ |
| *Srebp-1c* | 5’-CGTCTGCACGCCCTAGG-3’ | 5’-CTGGAGCATGTCTTCAAATGTG-3’ |
| *Fabp3* | 5’-CCCCTCAGCTCAGCACCAT-3’ | 5’-CAGAAAAATCCCAACCCAAGAAT-3’ |
| *Abca1* | 5’-GCTCTCAGGTGGGATGCAG-3’ | 5’-GGCTCGTCCAGAATGACAAC-3’ |
| *Zip7* | 5’-TCTGGGCCTACGCACTGGGG-3’ | 5’-TGGAGCAGAGAGCGGTGCCT-3’ |
| *Eef2* | 5’-ACGATGAGGCCGCCATGGGTAT-3’ | 5’-AAGTGGGCCTTTGGGGTCGC-3’ |
| *Il-6* | 5’-TTCCTCTCTGCAAGAGACTTCC-3’ | 5’-AGCATCAGTCCCAAGAAGGC-3’ |
| *Pygm* | 5’-AGCTGGAGCCTCACAAGTTC-3 | 5’-CAATGCGCTCAGCAATGACC-3’ |
| *Phkb* | 5’-GCTAGCATACAGGCGCATTG-3’ | 5’-TATCAGCCTGCCGCATGTAG-3’ |
| *Pgm2* | 5’-CATCCCGACCCCAATCTCAC-3’ | 5’-ATGTTTCGATCCCCGTCACC-3’ |
| *Gbe1* | 5’-ACTGCTTTGATGGCTTCCGT-3’ | 5’-AACCTTGACCCATTCCGTGG-3’ |
| *Zip1* | 5’-TGCATGTGACGCTTCAGTTC-3’ | 5’-TAAGCCAGCGTGATCTGCTC-3’ |
| *Zip13* | 5’-GCCAGCTTCCTTGTGAGCAA-3’ | 5’-GATAGCAAAGTCACCCACCTCA-3’ |
| *Zip14* | 5’-GGCTGGAGGACTTCAGTGTG-3’ | 5’-GGTGAGGCCAAGGCTAATGT-3’ |
| *Irs1* | 5’-CGATGGCTTCTCAGACGTG-3’ | 5’-CAGCCCGCTTGTTGATGTTG-3’ |
| *Irs2* | 5’-CTGCGTCCTCTCCCAAAGTG-3’ | 5’-GGGGTCATGGGCATGTAGC-3’ |
| *Insr* | 5’-ATGGGCTTCGGGAGAGGAT-3’ | 5’-GGATGTCCATACCAGGGCAC-3’ |
| Endogenous *Zip7* 5’UTR | 5’-GTCAAGTCGAGTCGTCTCTTGTTCC-3’ | 5’-CAGGTCACCATGACCCTCGTG-3’ |
| Exogenous pCMV*-Zip7* | 5’-TTGCCATGATGGTACTGATTGCC-3’ | 5’-AACCTTATCGTCGTCATCCTTGTAATCCA-3’ |
| Exogenous and endogenous *Zip7* | 5’-TGAAAGCATCTGGCATGGG-3 | 5’-TGGAGGCTATCGTGGGAGTG-3’ |
